# Optimizing CRISPR/Cas9 for the Diatom *Phaeodactylum tricornutum*

**DOI:** 10.3389/fpls.2018.00740

**Published:** 2018-06-06

**Authors:** Daniel Stukenberg, Stefan Zauner, Gianluca Dell’Aquila, Uwe G. Maier

**Affiliations:** ^1^Department for Cell Biology, Philipps-Universität Marburg, Marburg, Germany; ^2^LOEWE-Zentrum für Synthetische Mikrobiologie (SYNMIKRO), Philipps-Universität Marburg, Marburg, Germany

**Keywords:** CRISPR-Cas, genome editing, *Phaeodactylum tricurnutum*, diatom, off-target

## Abstract

CRISPR/Cas9 is a powerful tool for genome editing. We constructed an easy-to-handle expression vector for application in the model organism *Phaeodactylum tricornutum* and tested its capabilities in order to apply CRISPR/Cas9 technology for our purpose. In our experiments, we targeted two different genes, screened for mutations and analyzed mutated diatoms in a three-step process. In the end, we identified cells, showing either monoallelic or homo-biallelic targeted mutations. Thus, we confirm that application of the CRISPR/Cas9 system for *P. tricornutum* is very promising, although, as discussed, overlooked pitfalls have to be considered.

## Introduction

The characterization of cellular processes demands efficient manipulations of the genomes of the organisms of interest. Over the last years several genome editing systems were developed by which targeted mutations, either small insertions or deletions (indels) or single nucleotide variants (SNVs), can be introduced into specific regions of genomes (e.g., Sander and Joung, 2014). Especially for organisms showing no insertion of transformed DNA via homologous recombination, zinc finger nucleases or the TALEN (*Transcription activator-like effector nuclease*)- and CRISPR/Cas9 (Clustered regularly interspaced short palindromic repeats)- technology are powerful tools for genome editing (e.g., Zhang, 2014) and major breakthroughs in applying these technologies in diatoms, chromalveolate unicellular organisms, have been published recently. This includes TALEN as well as CRISPR/Cas9 (Daboussi et al., 2014; Hopes et al., 2016; Nymark et al., 2016; Mann et al., 2017; McCarthy et al., 2017; Serif et al., 2017). Although efficient genome editing activity was shown using both tools, CRISPR- and TALEN-specific advantages and disadvantages have been recognized. Especially less cloning efforts are needed for CRISPR/Cas9 compared to TALEN. However, several reports demonstrated a significant amount of off-target mutations when CRISPR/Cas9 was applied in model organisms (e.g., Fu et al., 2013, but see Kleinstiver et al., 2016).

In one of our projects we are interested in the response of diatoms to phosphate limitations in the environment. Recently published transcriptome and proteome data provided an in-depth knowledge on the expression of important proteins in respect to phosphate concentrations in the medium (e.g., Dyhrman et al., 2012; Yang et al., 2014; Zhang et al., 2016; Cruz de Carvalho et al., 2016). We used this data to extract putative factors from the diatom *Phaeodactylum tricornutum*. Here, we focused on Vtc2 (Phatr2| 35739^1^), a component of the vacuolar transport chaperone complex, and Pho4 (Phatr2| 23830^1^), a putative phosphate transporter. Although similar experiments in different labs indicated differences in transcriptional regulation, *vtc*2 and *pho*4 are, according to Yang et al. (2014), 2 times downregulated (*vtc*2) and 10 times upregulated for *pho*4 under phosphate limitations.

In our approach, we used the genes encoding Vtc2 and Pho4 as a proof-of-principle test for genome editing with the CRISPR/Cas9 technology in the diatom *P. tricornutum*. For that we designed an “easy to handle” vector, in which all components for CRISPR/Cas9 genome editing and selection for transformants were unified. Analyses of transformants showed that mono- and biallelic mutations can be generated easily with the use of this vector. In addition, we got no indications for off-target mutations at two predicted off-target sites of one CRISPR-Cas9 construct.

## Materials and Methods

### Cultivation of *P. tricornutum* and Biolistic Transformation

Cultivation of *P. tricornutum* (Bohlin, University of Texas Culture Collection, strain 646) was performed in artificial seawater with 50% salinity; 16,6 g sea salt (Tropic Marin Dr. Biener GmbH, Wartenberg, Germany) per liter distilled H_2_O, with f/2 supplements (Guillard, 1975) and buffered with 2mM Tris pH 8.0. For cultivation on solid media 1,3% of Agar-Agar Kobe I (Carl-Roth GmbH & Co. KG, Karlsruhe, Germany) was added. For non-induced conditions, NH_4_Cl (1.5 mM) was added as a nitrogen source. To induce *cas*9 the cells were spread on plates containing 0.89 mM NaNO_3_ as the sole nitrogen source. All media contain zeocin in a final concentration of 75 μg/ml for selection on transgenic *P. tricornutum* cell-lines. Cells were cultivated under constant illumination (80 μmol photons/m^2^s^1^). Liquid cultures were grown with agitation (150 rpm) on a horizontal shaker. Transformation of *P. tricornutum* was performed as described by Apt et al. (1996). Transformants were selected for genomic integration of ptCC9 on solid media under non-inducing conditions using NH_4_Cl (1.5 mM) as a nitrogen source and 75 μg/ml Zeocin. After 2 weeks of incubation, four to 25 Zeocin-resistant colonies were obtained and subjected to further analyses.

### Vector Construction

The plasmid ptCC9 (GenBank accession number: MH143578) is based on the plasmid pPha-DUAL-[2xNR] (GenBank: JN180664.1). The Cas9 coding region and the gRNA gene, the latter including the promoter/terminator regions, were amplified from pKSdiaCas9_sgRNA (Nymark et al. (2016), obtained from AddGene (AddGene ID: 74923).

**FIGURE 1:**
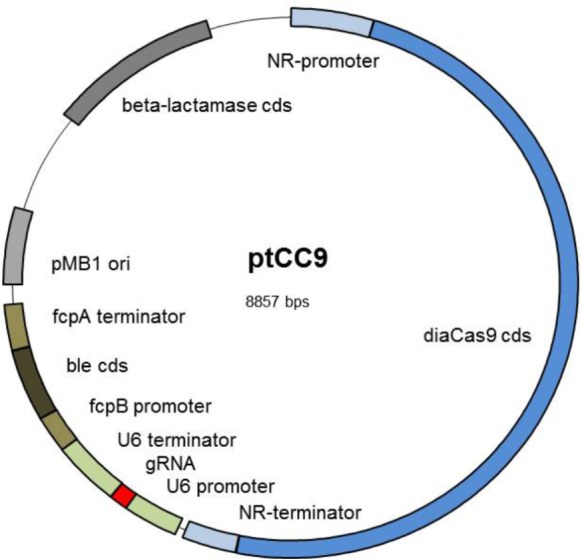
Scheme of the expression vector ptCC9: The vector ptCC9 is composed of the *diacas9* gene controlled by the NR promoter and the corresponding terminator and the gRNA expression cassette with U6 promoter and U6 terminator from *P. tricornutum*. Selection in *P. tricornutum* is enabled with the gene *ble*, which confers resistance to zeocin. This gene is controlled by the light induced promoter *fcpB* and terminator *fcpA*. For cloning purposes in *E. coli*, the plasmid harbors the high copy Ori from pMB1 and a beta-lactamase resistance cassette conferring resistance to ampicillin. Abbreviations are as follows: NR, nitrate reductase; ori, origin of replication; gRNA, guideRNA; cds, coding DNA sequence.

In order to eliminate two *Bsa*I recognition sites from pPha-DUAL-[2xNR], the Q5^®^ Site-Directed Mutagenesis Kit (New England BioLabs) was applied using the primers 5_5′_Muta1 and 6_3′_Muta1 for the first and 7_5′_Muta2 and 8_3′_Muta2 for the second recognition site (all primers are listed in Supplementary Table 1). The two CRISPR/Cas9 specific parts, a codon optimized version of *S. pyogenes cas9* (*diacas9*), and the gRNA expression cassette, including the U6 promoter and U6 terminator, were amplified via PCR from pKSdiaCas9_sgRNA (primers 4_5′_NdeI_U6Prom and 3_3′_AccIII_U6Term, and in case of diaCas9 23_GA_5′_Cas9 and 24_GA_3′_Cas9). Both fragments were cloned into pPha-DUAL-[2xNR] using Gibson assembly. The vector was PCR amplified using primers 13_GA_3′pPhaNRdual_NdeI and 14_GA_3’pPhaNRdual_AccIII for the cloning of the gRNA cassette and in a second step the vector was PCR amplified using primers 25_GA_5′_Vektor and 26_GA_3′_Vektor for cloning of *diaCas9*, leading finally to ptCC9 (**Figure 1**).

### Selection and Cloning of Spacer Sequences

Target sites for the CRISPR/Cas9 system were identified using the web based software Benchling^2^ (Benchling [Biology Software], 2017). For each targeted gene, two gRNAs were cloned, one with a high on-target (Doench et al., 2016) and the other with a high off-target score (Hsu et al., 2013) (For primer sequences see Supplementary Table 2). Selected guideRNAs were cloned into the s*gRNA* of ptCC9 using annealed synthetic oligomers and *Bsa*I digested ptCC9.

### Induction of CRISPR/Cas9 and Screening of Targeted Mutations

To induce diaCas9 expression, zeocin resistant diatoms were transferred to agar plates containing 0,89 mM NaNO_3,_ incubated for 7 days and either the *vtc*2 or *pho*4 genes were inspected for mutations via colony PCR and sequencing of the amplicons (Moog et al., 2015). In the second step of screening, clones showing sequence “ambiguities” in the target region (based on sequencing of PCR products) were grown in liquid culture and spread on agar plates to obtain single colonies. These colonies were analyzed as described above. For step III of the screening, the PCR products of step II were cloned into pJET1.2/blunt Cloning Vector (Thermo Fisher Scientific) and transformed into *E. coli top10 (Invitrogen)*. Plasmids were isolated and subsequently sequenced using vector specific primers.

### Calculation of CRISPR/Cas9 Activity Factor (CAF)

Ab1 files obtained from sanger sequencing were uploaded into the Minor Variant Finder Software v.1.1 (Applied Biosystems, Thermo Fisher Scientific) and converted into csv files. Peak intensities in rows where base call is shown were extracted for subsequent analysis. The following calculation was carried out for the regions from 50 bp upstream up to 100 bp downstream of the expected DSB (-1 defined as position 4 bp upstream of the PAM and +1 defined as position 3 bp upstream of the PAM):

$$CAF=\frac{\text{signal intensity of wild type base}}{\text{total signal intensity}}$$

The CAF for each position was displayed in a scatterplot. The arithmetic mean of the regions for -50 up to -10 and +1 up to +100 were calculated. The region from -9 up to -1 was excluded because we observed in some cases ambiguities in the regions slightly upstream of the expected DSB, presumably caused by deletions. The arithmetic mean of the negative region was subtracted from 1 to compensate for sequence ambiguities caused by the difference between the sequencing process and the CRISPR/Cas9 system. The obtained difference is added to the arithmetic mean for the positive region, which yields the corrected mean CAF (cmCAF) for each induced clone analyzed in step I.

### Search for Off-Targets

Exemplarily, off-target sites of Phatr2| 42441^1^ and Phatr2| 41088^1^ (see above) were inspected. Putative off-target sites were predicted by Benchling (Benchling [Biology Software], 2017; see footnote 2). To do so the coding regions of both genes, which have a predicted, most-likely off-target site, were PCR amplified from five independent VTC-158 clones and subsequently sequenced.

## Results

### Cloning Strategy for an Expression Vector

In order to design a manageable basic vector for the CRISPR/Cas9 approach in *P. tricornutum*, we wanted to integrate the genes encoding for Cas9 and gRNA (guide RNA) into one plasmid, which can additionally be selected for integration into the genome after transformation of the diatom. We used the vector pPha-DUAL-[2xNR] as a template, which had previously been designed by us, to express two coding regions (GenBank: JN180664.1). In addition, cells transformed with this plasmid can be selected through an ampicillin (*E. coli*) or zeocin (*P. tricornutum*) resistance. In the first step, we inserted the coding region of Cas9, for which the Winge lab had previously designed a codon-optimized version for diatoms (obtained from the plasmid pKS diaCas9_sgRNA, Addgene^3^, Nymark et al., 2016). This gene was fused to the nitrate reductase (NR) promoter and terminator regions. Although it is known the NR promoter is not completely inactive under non-inducing conditions, its activity can at least be strongly enhanced (Chu et al., 2016), so that the transcription of the downstream genes (here Cas9) is assured in media with nitrate as the sole N-source. Next we inserted a coding region for the *gRNA*, flanked by *P. tricornutum’s* U6 promoter and the corresponding 3′ region, using pKSdiaCas9-sgRNA as a template. This basic vector, called ptCC9, is shown in **Figure 1** and differs from pKS diaCas9-sgRNA in two features: the plasmid possesses a zeocin resistance gene (*ble*, **Figure 1**) and the *ca*s9 gene is under the regime of the NR promoter.

### Induction of CRISPR-Modules and Characterization of Mutations

In order to test the designed expression vector and the possibilities of the CRISPR/Cas9 system in our hands, we wanted to target two genes, *vtc*2 and *pho*4. Both genes are transcriptionally regulated according to the phosphate concentration in the medium (according to Yang et al., 2014). We have recently shown that Vtc2 is a protein of the vacuolar membrane (Schreiber et al., 2017).

To design the oligo nucleotides used for cloning the 5′ part of the *gRNA*, we applied algorithms described in Doench et al. (2016) and Hsu et al. (2013), both available from Benchling (Benchling [Biology Software], 2017; see footnote 2). Using these algorithms potential on- and off-target regions within gene sequences were predicted. We decided to tackle the genes by using two different *gRNA*s, one which covers a region predicted to have a high on-target score, the other which covers a region with a high off-target score. A high on-target score indicates that a region is well suited for creating mutants, a high off-target score indicates that it is probable for a construct to create off-target mutations. For *vtc*2, we used the nucleotide positions 141–160 (VTC2-158, high on-target) and 255-274 (VTC2-272, high off-target) and nucleotide positions 34–53 (PHO4-51, high off-target) and 963–982 (PHO4-980, high on-target) for the *pho4* gene (Supplementary Table 2). These sequences were cloned as the 5′ end of the g*RNA* and the resulting vectors were transformed. Selection of transformants was done according to their zeocin resistance, which is controlled by the *fcp*B promoter. For increased *cas*9 expression zeocin resistant clones were transferred to agar plates supplemented with nitrate as the sole N-source.

**FIGURE 2:**
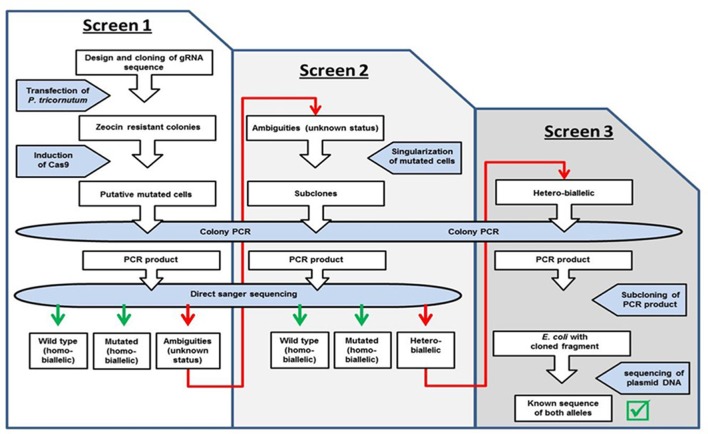
Workflow. Indicated are the different steps of the screen for homo- and biallelic mutations.

We expected a high efficiency of the CRISPR/Cas9 system and therefore analyzed a manageable number of zeocin resistant clones after induction of the Cas9 module (ten PHO4-51, five PHO4-980, four VTC2-158, and six VTC2-272 clones), as shown in step 1 of the workflow in **Figure 2**. In order to get a first impression of the activity of the system, we isolated DNA from the “clones” and amplified the region targeted by the CRISPR/Cas9 module. At this point in the screening process, direct sequencing of the PCR products can show one of three different scenarios: either a sequence identical to the wild type, or a non-wild type sequence indicating a mutation, which might reflect either a “true” homo-biallelic mutation or a hetero-biallelic one in which, beside a SNV in one allele, a deletion in the other impairs amplification via PCR of this allele (in the further text referred to as a homo-biallelic mutation, but discussed below), or a sequence with sequence ambiguities following the position of the expected double strand break (DSB) due to overlapping peaks, most probably caused by different indel/SNV mutations in different cells of a “clone” or chromosomes (in the further text referred to as ambiguities).

**FIGURE 3:**
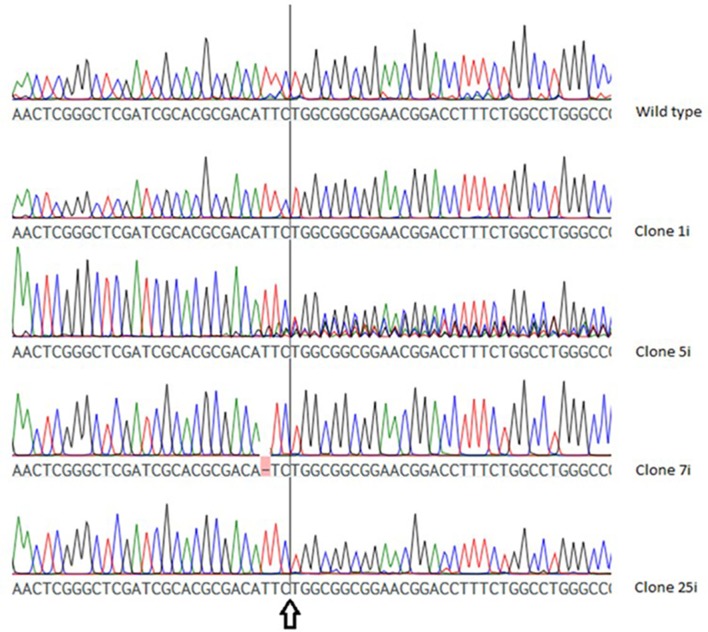
Chromatogram of the wild type *vtc*2 gene and clones expressing ptCC9 with nucleotides 141-160 as the spacer of the *gRNA* gene (VTC2-158). The arrow indicates the position of the expected DSB.

As shown in **Figure 3**, the chromatogram of two VTC2-158 clones (VTC2-158-1i, VTC2-158-25i) showed no deviation to that of the wild type, whereas in case of clone VTC2-158-7i one base pair was deleted upstream of the position of the expected DSB. The chromatogram of clone VTC2-158-5i showed pronounced ambiguities downstream of the DSB. The results of the first step of the screening process for CRISPR/Cas9 induced mutations in *vtc*2, which indicated no mutations in clones VTC158-1i and -25i, and one biallelic (mutated) genotype of one bp deletion in clone VTC2-158-7i. Clone VTC2-158-5i could be composed of different cells, which have different alleles of the *vtc*2-gene. In the case that all cells have a clonal origin stemming from the mutagenic event, a monoallelic or a hetero-biallelic mutation might also be possible. To find an answer this “clone” needs to be further characterized using the second step of the screening process (**Figure 2**).

**FIGURE 4:**
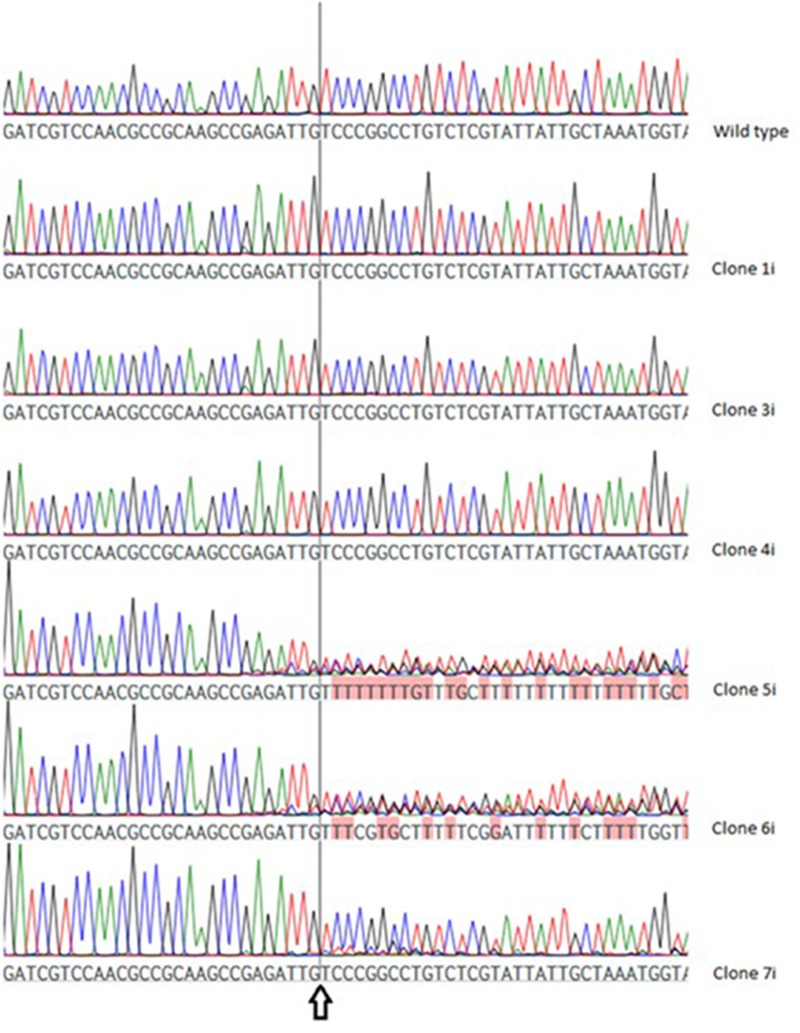
Chromatogram of the wild type *vtc*2 gene and clones expressing ptCC9 with nucleotides 255-274 as the spacer of the gRNA gene (VTC2-272). The arrow indicates the position of the expected DSB.

In VTC2-272, three of the six investigated clones (VTC2-272-1i, -3i and -4i) showed no differences when compared to the wild type sequence, whereas in the chromatogram of the others (VTC2-272-5i, -6i and -7i) differentially pronounced ambiguities were detected (**Figure 4**).

In the experiments with PHO4-51 (Supplementary Figure 2) four of the obtained clones (-4i, -7i, -12i, -14i) showed the wild type sequence, whereas sequencing of six further clones (-3i, -5i, -6i, -9i, -11i, -13i) resulted in a chromatogram with different intense ambiguities downstream of the expected DSB.

In the experiments with PHO4-980, all of the investigated clones expressed the wild type gene sequence (not shown).

As described, we observed differently pronounced ambiguities in some of our experiments. We assume that the “strength” of the ambiguities correlate with the amount of non-wild type sequences and therefore can help to decide which clones should be analyzed further. Because of this, we quantified the extent of the ambiguities and developed a formula of which the result represents the ‘strength’ of CRISPR/Cas9 activity. To do so, the CRISPR/Cas9 activity factor (CAF), which represents the ratio of the signal intensity of the base sequence (expected for a wild type) to the total signal intensity, was calculated for each position. A value of 1,0 indicates a “clean” wild type peak without any ambiguity, while a value of 0,0 indicates the absence of a wild type sequence. A defined region upstream of the expected DSB was used to compensate for sequence ambiguities caused by the sequencing process rather than by CRISPR/Cas9 activity, finally giving the corrected mean CAF (cmCAF).

**FIGURE 5:**
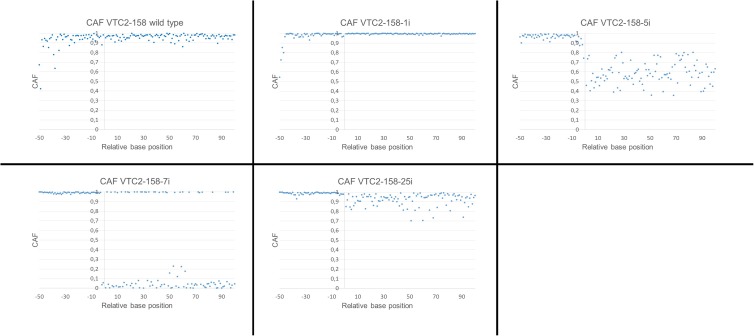
Scatterplot of the calculated CAFs for VTC2-158. The ordinate shows the position of the peaks relative to the position of the expected double strand breaks. –1 is defined as the position 4 bp upstream of the PAM and +1 is defined as the position 3 bp upstream of the PAM. The abscissae represent the calculated CAF for each position.

This calculation was applied to both Vtc2 constructs. A cmCAF of 1,03 and 1,05 was obtained, for VTC2-158-1i and for the wild type sequence respectively, which indicates no CRISPR/Cas9 activity. The calculated cmCAF for VTC2-158-5i is 0,61. This fact, combined with the drop of the CAFs following the position of the expected DSB seen in the scatterplot (**Figure 5**), supports the presence of CRISPR/Cas9 activity, as indicated by the ambiguities seen in the chromatogram. With VTC2-158-7i, we had already identified an one bp deletion with no ambiguities (**Figure 3**). In the scatterplot this is shown as two distinct populations of dots with CAFs either close to 1,0 or close to 0,0. This is the expected result for a “clean” indel mutation because statistically every fourth base of a shifted sequence matches the base expected for a wild type sequence. The cmCAF calculated for VTC2-158-25i is 0,93. A slight drop of the CAFs can be seen in the region following the double strand break, similar but weaker than what was seen in VTC2-158-5i’s results. This might be evidence of minor CRISPR/Cas9 activity in this clone, which could not be seen in the chromatogram. As our primary goal of isolating mutants and promising candidates had already been achieved, VTC2-158-25i was not taken into consideration for further steps of the screening process. In the case of experiments which lead exclusively to seemingly minor or not detectable CRISPR/Cas9 activities, applying our here described approach might indicate mutation events hidden in the chromatograms. The clones -1i (cmCAF = 1,01), -3i (cmCAF = 0,98), -4i (cmCAF = 1,00), and the wild type control (cmCAF = 0,99) gave no evidence for any CRISPR/Cas9 activity in the experiments with VTC2-272. The results for VTC2-272-5i (cmCAF = 0,30) and VTC2-272-6i (cmCAF = 0,50) support the impression of a strong CRISPR/Cas9 activity as showed in the chromatograms. In VTC-272-7i’s (cmCAF = 0,87) case the calculation might support the assumption based on the chromatogram’s results (**Figure 4**), which show a slight CRISPR/Cas9 activity was induced in this clone (for scatter plots see Supplementary Figure 1, for chromatograms see **Figure 4**). This clone was not analyzed further for the same reasons as VTC2-158-25i.

In conclusion, we observed a high efficiency of the CRISPR/Cas9 system in both Vtc2-approaches (50% show significant CRISPR/Cas9 activity) and in the PHO4-51 experiment (60%). However, gene- and gRNA-specificities might be a potential source of problems, as it might have been in the PHO4-980 experiment, in which no mutated cells could be selected.

### Screening for and Validation of Biallelic Mutations

Next we wanted to characterize clones that showed ambiguities in the chromatograms in order to decide if the characterized cellular material was genetically identical or composed of cells in which the genome editing system worked differently. Additionally, we cannot dismiss the possibility that the clones characterized in step I of the screening were not of monoclonal origin. This was done in step II (**Figure 2**) with clones 5i of VTC2-158, 5i of VTC2-272, and 3i of PHO4-51. We also re-analyzed the predicted biallelic mutation of clone 7i of VTC2-158. For each clone, we first propagated the cells in liquid medium and plated dilutions onto agar plates to get single colonies. The genomic DNA was isolated from single colonies and the genomic region spanning the predicted mutations was amplified and sequenced.

Here we show the results of seven single “sub”-clones of VTC2-158 obtained from 5i (VTC2-158-5iA to VTC2-158-5iG) and four from clone 7i (VTC2-158-7iA to VTC2-158-7iD). In VTC2-272’s case the sequences of two clones of 5i are shown (VTC2-272-5iA, VTC2-272-5iB). For PHO4-34 two colonies obtained from clone 3i (PHO4-51-3iA and B) and two from 13i (PHO4-51-13iA and B) were investigated in detail.

As shown in Supplementary Figure 3, sequencing of the VTC2-158-5i clones indicated ambiguities in the *vtc*2 sequence of the clones A, B, D, E and G, whereas the clone C showed the wild type sequence. In VTC2-158-5iF’s case, two PCR products with different lengths were obtained. This clone was analyzed further as described in the next chapter.

The results obtained from the sub-clones of VTC2-158-7i were promising; all investigated cells had a confirmed 1 bp deletion upstream of the DSB (Supplementary Figure 4).

The two analyzed clones obtained from 5i of VTC2-272 showed a cytosine and a thymidine insertion upstream of the DSB (Supplementary Figure 5).

In the *pho*4 gene, we identified a two bp deletion in clone 3iA and 3iB (3iA without ambiguities), whereas from clone Pho4-51-13i colonies were identified, which showed a 6 bp insertion (PHO4-51-13iA) or a one bp deletion (13iB) (Supplementary Figure 6).

In case of PHO4-980, all of the investigated clones expressed the wild type gene sequence (not shown).

In conclusion, we isolated three different biallelically mutated strains (VTC2-158-7iA, VTC2-272-5iA, VTC2-272-5iB) for *vtc*2 and additional three (PHO4-51-3iA, PHO4-51-13iA and PHO4-51-13iB) for *pho*4. However, it should be noted that in PHO4-51-13iA’s case the six bp insertion did not cause a frameshift downstream of the mutated site.

In order to evaluate the capacity of the CRISPR/Cas9 system acting in diatoms in more detail, we performed a third step of screening (step III, **Figure 2**), with the aim to characterize clones not expected to be homo-biallelic.

### Characterization of Non Homo-Biallelic Mutations

The already identified non homo-biallelic mutants can have either one wild type allele and a mutated one (monoallelic), or different mutations in each allele (hetero-biallelic). This difference in the genotype cannot be determined by the chromatograms presented here. For further characterization of non homo-biallelic mutant strains we exemplarily studied the genotype of two clones, VTC2-158-5iE and VTC2-158-5iF. In VTC2-158-5iE’s case, we sub-cloned the PCR product of the screening process described above and sequenced six clones. Four of them showed a 11 bp deletion in the *vtc*2 gene, in the remaining clones the wild type sequence was cloned, which indicates a mono-allelic mutant strain (Supplementary Figure 7). As mentioned above, we got two different PCR products by analyzing possible mutations in the *vtc*2 gene of VTC2-158-5iF. Both fragments were sub-cloned and sequenced. Three clones showed the wild type sequence of the longer PCR product, whereas in smaller fragments three investigated clones had the same 209 bp deletion. This result also indicates a mono-allelic genotype (Supplementary Figure 8).

### Searching for Off-Targets

The CRISPR/Cas9 system can induce additional mutations in genomic regions besides the targeted mutations of interest (e.g., Fu et al., 2013; Iyer et al., 2015). These so-called off-target mutations might affect the phenotypical description of a mutant strain, especially in case the off-target mutation is located in a coding or a non-coding region with regulatory functions of the genome. Although it is known that only the complete genome sequence of the mutant is perfectly indicative for the occurrence of off-target mutations, we investigated putative, most likely off-target sites in VTC2-158 clones to get a first impression on side effects. According to the prediction algorithm used (Hsu et al., 2013, provided by Benchling), the two most likely off-target sites in coding regions are present in the genes Phatr2| 42441^1^ and Phatr2| 41088^1^. Both putative off-target regions differ in three nucleotides from the sequence at position 141-160 of the *vtc*2 gene (Supplementary Table 3).

We isolated genomic DNA from VTC2-158-5iD, -5iE, -5iF,-5iG and from the bi-allelic strain VTC2-158-7iB, and amplified the coding regions of proteins with Phatr2| 42441^1^ and Phatr2| 41088^1^, showing the two most likely off-target sites, and sequenced the PCR-products. Fortunately, all of the investigated clones showed the wild type sequence at both putative off-target positions (Supplementary Figures 9, 10).

## Discussion

Several methods have been developed for gene editing; all having adv(e.g., Daboussi et al., 2014; Ma and Liu, 2015). Consequently, it is not possible to predict at the moment which method will establish itself in the future, especially because there are developments being made in RNA editing and programmable base editing without DNA cleavage in genomic DNA (Cox et al., 2017; Gaudelli et al., 2017).

In the approach presented here, we were interested in establishing, simplifying and testing the CRISPR/Cas9 system for *P. tricornutum*, but not in a physiological characterization of mutant strains, which will be carried out in the future. For that, we targeted two genes, which encode proteins involved in phosphate homeostasis in *P. tricornutum*. These are a vacuolar protein, Vtc2, and a putative phosphate transporter, Pho4, both transcriptionally regulated in a phosphate-limited environment (Yang et al., 2014). We decided to test the CRISPR/Cas9 system in the diatom *P. tricornutum* for several reasons. One reason was that the pioneering work of the Winge lab had already demonstrated that the application of the CRISPR/Cas9 technology is generally possible in *P. tricornutum* (Nymark et al., 2016). A further reason was that the cloning efforts for gene editing approaches via CRISPR/Cas9 are markedly lesser in comparison to other systems such as TALEN. We designed a vector to simplify the process of cloning and transformation to create mutants. As in the work of the Winge lab, the relevant genes for CRISPR/Cas9 genome editing were cloned into one vector (Nymark et al., 2016). However, to increase the efficiency of transformation (and selection) we also equipped the vector with a zeocin resistance gene cassette, so that only one vector, instead of two, has to be transformed. Another characteristic of the vector ptPCC9 is the expression of the Cas9 via the nitrate reductase promoter. Although it is known that this promoter is not totally shut-off under non-inducing conditions, its activity can be up-regulated using nitrate as the sole N-source (Chu et al., 2016). Thus, a vector has been created for *P. tricornutum*, with species-specific adaptations, which are similar to a reported vector used for editing in the diatom *Thalassiosira pseudonana* (Hopes et al., 2016).

As specified later in more detail, one disadvantage of the CRISPR/Cas9 system might be side reactions leading to so called off-target mutations (Iyer et al., 2015; Zhang et al., 2015). Since every *gRNA* is supposed to have different putative off-targets, we decided to tackle each gene of interest at two different positions, as seen in other published CRISPR/Cas9 experiments (e.g., Nymark et al., 2016). The idea behind this is that in experiments leading to two different mutations in the same gene, the off-targets should be different. Thus, in case an identical phenotype can be observed in both strains, one can assume it’s not caused by an off-target mutation.

We used a two-step screening procedure to characterize putative mutants induced by the CRISPR/Cas9 system. In addition, for clones assumed to be affected in only one allele (monoallelic mutation) or by different mutations in both alleles (hetero-biallelic strain), a further screening step was applied (**Figure 2**).

We first transformed the cells with the plasmids designed to induce mutations at two different targets, within the genes for *vtc*2 and *pho*4. Zeocin resistant colonies, expected to represent positively transformed clones were isolated and subjected to nitrate induction plates in order to increase the *cas9* expression. After one week of incubation, the colony’s DNA was analyzed for indels or SNVs by comparing the sequence to the corresponding wild type gene. At this step of screening for mutants we got three different types of colonies: (i) colonies with no mutations, (ii) colonies showing ambiguities, which might indicate that the CRISPR/Cas9 system induces genome editing events that differ in respect to the alleles or cells, and (iii) one clone (VTC2-158-7i, **Figure 3**) having a mutation, in which no additional ambiguities were detected. In this case, it can be postulated that in the founder cell of the clone a homo-biallelic strain was created, which also indicates that the NR promoter of the Cas9 gene is not completely inactive under non-inducing conditions. In colonies with ambiguities in the targeted sequence, one cannot be sure if the investigated colony is either of genetically monoclonal origin (as in case of VTC2-158-7i) or has indels/SNVs in one allele only. In order to quantify the extent of the ambiguities we developed a calculation of the cmCAF to additionally analyze the chromatograms. As shown, this correlates accurately with the data observed in the chromatograms and is an especially important step if minor or no evidence of CRISPR/Cas9 activity is found in the experiments after induction of the *cas9* in step I of the screening.

We performed a second screening step for mutants with selected clones obtained in the first screening process. In order to confirm the finding that one homo-biallelic mutation was already selected in the first step of our screening, we also included the clone VTC2-158-7i in the second screening step (**Figure 2**).

In any case, the analyses of the clones stemming from the first step of our screening indicated a high efficiency of the CRISPR/Cas9 editing system in three out of four experiments. To explain clones that display a zeocin-resistance and are CRISPR/Cas9 negative, one has to consider that a transformed plasmid is integrated into the genome as a linear molecule and the double strand break for linearization occurs randomly. Thus, not many variants for “functional” integration of the “plasmid” exist, because a high percentage of the plasmid ptCC9 is covered by the promoters/terminators and the genes of Cas9 and gRNA (**Figure 1**). However, as shown here, by screening several clones one can obtain zeocin-resistant clones with mutations in the target region.

To select colonies with a monoclonal origin, we incubated cells showing ambiguities or the putative homo-biallelic mutation (VTC2-158-7i) in liquid culture and diluted it in such a way that single colonies were obtained when re-plating on agar plates. We isolated genomic DNA of single clones next and amplified the target regions of the CRISPR/Cas9 system. After direct sequencing the PCR products, ambiguities or the wild type sequence were detected in many chromatograms. However, as indicated by a non-wild type sequence with no additional ambiguities, five clones highlighted different homo-biallelic mutations (VTC2-272-5iA, VTC2-272-5iB in case of VTC2-272 (Supplementary Figure 5), and PHO4-51-3iA, PHO4-51-13iA, PHO4-51-13iB) (Supplementary Figure 6). In addition, it was confirmed that clone VTC2-158-7i has a homo-biallelic mutation. These results illustrate the importance of the second step of the screening for clones that were not shown to be homo-biallelic in the first screen. This allows biallelic mutations to be isolated even though only ambiguities were detected in the first step. Moreover, this outcome suggests that the activity of the CRISPR/Cas9 module might differ in individual cells and therefore an apparently clonal origin of cells needs to be further investigated.

As mentioned, cells for which ambiguities could be determined in step II of the screening might have either a monoallelic mutation or contain different mutations in each allele of the targeted genes (hetero-biallelic). However, from the chromatograms it is impossible to decide which genotype is the correct one. Especially in case of the latter, a hetero-biallelic mutation affecting the reading frame of both alleles is useful for a phenotypical description of strains. Thus we studied the genotype of two ambiguity-strains (VTC2-158-5iE and VTC2-158-5iF), obtained from step II of the screening, in a third step of screening (**Figure 2**), analyzing 12 sub-clones altogether. This resulted in a monoallelic genotype being determined for both ambiguity-strains; however, investigation of further ambiguity-clones might result in the identification of hetero-biallelic mutations.

There are several pitfalls in the determination of mutations created via the CRISPR/Cas9 technology, one of them being the detection of putative homo-biallelic mutations. As can be observed in the results of the analysis of clone VTC2-158-5iF, CRISPR/Cas9 can induce large deletions or, as reported elsewhere (Nymark et al., 2016), insertions. Therefore, by analyzing the alleles of the targeted genes via PCR, one cannot exclude that large deletions/insertions have occurred, and if the resulting amplicons originated from both alleles or one single allele. However, in the case of a single mutation in one allele (detectable in the PCR product) and a deletion/insertion in the other (not detectable via PCR), the resulting genotype (a hetero-biallelic mutation) might not affect the phenotypical description of a strain in most cases because the coding regions of both alleles are affected. However, such an undetected deletion (if large enough) could have tremendous effects on all downstream analyses when affecting an up or downstream located gene. To minimize this, the use of two different gRNAs per analyzed gene is recommended, as it is to sniff putative off-target effects (discussed above). Ideally, one can use clear hetero-biallelic clones where both alleles could be identified as mutated for downstream analyses.

A major concern when applying the CRISPR/Cas9 system is off-target mutations (e.g., Fu et al., 2013). Currently, the best strategy to detect these unwanted mutations is a whole genome sequencing approach, in which the genomic sequence of the mutated strain is compared to that of the wild type. Whereas the sequencing part is not a real challenge, the assembly of the genome of the mutant and alignment with the wild type sequence is labor intensive. Thus, alternative approaches are required. A relatively straightforward alternative was used here; the most susceptible off-target sites in coding regions were sequenced. Although this approach only offers a glimpse into the presence or absence of mutations at likely off-target sites, it is manageable in all labs. We used four clones showing ambiguities in *vtc*2 (VTC2-158-5iD, -5iE, -5iF, and -5iG) and one homo-biallelic strain (VTC2-158-7iB) for this approach. We chose VTC2-272 to search for off target sites since it has a lower off-target score than VTC2-158 (99,9 and 98,8 respectively) and is therefore more likely to lead to off-target mutations. Although sequencing of the two potential target sites in five different strains might not be of the highest statistical relevance, the lack of any mutations in the potential off-target sites could indicate that *P. tricornutum* is not very susceptible for side effects of the CRISPR/Cas9 system. Regardless, we cannot dismiss the possibility that the CRISPR/Cas9 system interfered with other genomic regions. As the latter might occur at a time after induction, elimination of the CRISPR/Cas9 module after the generation of mutations is desirable, for which strategies have to be developed. As both components of the CRISPR/Cas9 system, *gRNA* and Cas9, need to be present in the nucleus simultaneously, the controlled and temporally limited expression of one component could decrease the probability of off-target mutations compared to a constitutive expression of all components. Promoters, which can be shut-off easily would be perfect to achieve this, which does not perfectly apply to the nitrate reductase promoter used in this experiment.

Finally, we would like to discuss a further, often ignored, pitfall in applying CRISPR/Cas9 in diatoms. The creation of a strain meant to be mutated starts with the transfection of a plasmid, which introduces the necessary genes into the genome of the recipient. For diatoms, the integration of foreign DNA into the genome is random and cannot be controlled. In addition, sometimes diatom clones express a foreign gene in higher amounts than the others do, which is caused by multiple integrations of the transferred plasmid according to our experience in recombinant antibody expression (Hempel et al., 2017). Therefore, the initial integration of a CRISPR/Cas9 vector in the genome might occur in coding or regulatory regions of the genome, which can affect the phenotype of clones containing biallelic mutations. Although using only one plasmid for CRISPR/Cas9 mutations instead of two might reduce these adverse effects, one should still determine the quantity and the loci of the integration events into the genome carefully.

## Conclusion

We can confirm the efficiency of the CRISPR/Cas9 system in the diatom *P. tricornutum*. By optimizing the vector, encoding a CRISPR/Cas9-module we simplified the application in this diatom and induced mono- and biallelic mutations, which were analyzed in a three-step screening process. Our data suggest that off-target mutations might be rare, at least at the most likely sites in coding regions. In any case, by additional investigations on the integration of the CRISPR/Cas9 plasmid into the genome and using two different target sites to generate single gene mutations, an intelligent complementation strategy could finally be created which highlights successfully mutated clones, which can be tested for biological effects.

## Author Contributions

UM conceived this study together with DS and SZ. DS, SZ, and GD did the experimental work. The manuscript, which all authors read and approved, was written by UM.

## Conflict of Interest Statement

The authors declare that the research was conducted in the absence of any commercial or financial relationships that could be construed as a potential conflict of interest.

## Footnotes

1 https://genome.jgi.doe.gov/Phatr2/Phatr2.home.html

2 https://benchling.com

3 https://www.addgene.org/

## Abbreviations

PCR: polymerase chain reaction;
