# Supplementary material for: Optimizing CRISPR/Cas9 for the Diatom Phaeodactylum tricornutum
Source: Front Plant Sci. 2018 Jun 6;9:740. doi: 10.3389/fpls.2018.00740 (PMC5998643; doi:10.3389/fpls.2018.00740)

Supplementary Material

**Optimizing CRISPR/Cas9 for the diatom *Phaeodactylum tricornutum***

**Daniel Stukenberg^1+^, Stefan Zauner^1+^, Gianluca Dell`Aquila^1^**, **Uwe G. Maier ^1, 2^***

*** Correspondence:** Prof. Dr. Uwe G. Maier: [maier@biologie.uni-marburg.de](mailto:maier@biologie.uni-marburg.de)

**Suppl. Table 1:**

Primers used in this study. Lowercase letters represent 5`overhangs of the primers used for cloning purposes.

| 3_3´_GA_AccIII_U6Term | tccggaGCAGAAAAGTTCGTC | Amplification of gRNA cassette |
| --- | --- | --- |
| 4_5´_GA_NdeI_U6Prom | catatgGTTGGCTCGGAAGTTGGTG | Amplification of gRNA cassette |
| 5_5´_Muta1 | tCCACGCTCACCGGCTCCAG | Removal of *Bsa*I site 1 |
| 6_3´_Muta1 | TCTCGCGGTATCATTGCAGCACTGG | Removal of *Bsa*I site 1 |
| 7_5´_Muta2 | aGTCTCCCTATAGTGAGTCG | Removal of *Bsa*I site 2 |
| 8_3´_Muta2 | GGCCTCGAGAAAACTCATC | Removal of *Bsa*I site 2 |
| 9_3`_Sequencing | CGTTATCCCCTGATTCTGTGG | Sequencing primer to verify *Bsa*I site removal |
| 13_GA_3´pPhaNRdual_NdeI | caacttccgagccaacCATATGCTAAGTTCTAACGCAGCTTAG | Amplification of pPha-DUAL-[2xNR] for cloning of gRNA cassette |
| 14_GA_5´pPhaNRdual_AccIII | cgacgaacttttctgcTCCGGAGAGATTCAAAATTCAATCTG | Amplification of pPha-DUAL-[2xNR] for cloning of gRNA cassette |
| 15_5´_Seq_gRNA | AGGAAGTGAAACTCGGTTCG | Sequencing primer for gRNA sequence |
| 23_GA_5`_Cas9 | agtcaccacttgtgcgaacgATGGCCTCTAGAATGGAC | Amplification of *diacas9* |
| 24_GA_3`_Cas9 | gctagtcttaaagtaaattgTTACTTTTTCTTTTTTGCCTG | Amplification of *diacas9* |
| 25_GA_5`_Vector | CAATTTACTTTAAGACTAGCGCAATTTTTTTC | Amplification of pPha-DUAL-[2xNR] + gRNA for cloning of gRNA cassette |
| 26_GA_3`_Vector | CGTTCGCACAAGTGGTGAC | Amplification of pPha-DUAL-[2xNR] + gRNA for cloning of gRNA cassette |
| 35_5`_Cas9_Seq | TGATATTGATTACAAAGACG | Sequencing primer for cloned *diacas9* in ptCC9 |
| 36_5`_Cas9_Seq | TCCTTTCTGCCCGACTCAGC | Sequencing primer for cloned *diacas9* in ptCC9 |
| 37_5`_Cas9_Seq | AAGGAGCTTCCGCGCAATCC | Sequencing primer for cloned *diacas9* in ptCC9 |
| 38_5`_Cas9_Seq | TATTAAAAAGGGCATTCTCC | Sequencing primer for cloned *diacas9* in ptCC9 |
| 39_5`_Cas9_Seq | CAACTACCATCACGCGCACG | Sequencing primer for cloned *diacas9* in ptCC9 |
| 40_5`_Cas9_Seq | CGCTTCTGCCGGCGAACTCC | Sequencing primer for cloned *diacas9* in ptCC9 |

| 42_3`_Vtc2 | TCTAGAATTGGATTCCCTGTCGTCCTTCAAG | Amplification of *vtc2* for screening of mutants |
| --- | --- | --- |
| 43_5`_Vtc2 | GAGCTCATGATTCACAGCCGTACTCTCCCACG | Amplification of *vtc2* for screening of mutants |
| 44_5`_Vtc2_KO_Seq | TCCCACGTCTTTGCCAAACG | Sequencing primer for detection of mutations in *vtc2* |
| 48_5`_Pho4_EcoRI | gaattcATGAGCGTTGACATGAGTCCTTTTCTTTGG | Amplification of *pho4* for screening of mutants |
| 49_3`_Pho4_XbaI | tctagaTCAGGCAGAGGGGGAAAAAGC | Amplification of *pho4* for screening of mutants |
| 50_3`_Pho4_KO_Seq | AGAGCAAGGACGTGAACATACC | Sequencing primer for detection of mutations in *pho4* |
| 51_5`_Pho4_KO_Seq | TTGCGCCAAGAAGCCTGAGG | Sequencing primer for detection of mutations in *pho4* |
| 67_5`_42441_Off-Target | GCTGCTGCGTTTGACGTACC | Amplification of potential off-target site 42441 for screening of mutants |
| 68_3`_42441_Off-Target | GGCCATCCCGCTTGCAAAGC | Amplification of potential off-target site 42441 for screening of mutants |
| 69_5`_42441_Seq | TGTCGAATTCCAACACATCG | Sequencing primer for detection of mutations in potential off-target site 42441 |
| 70_5`_41088_OFf-Target | TGCGAGCGTGCTCTCATTGC | Amplification of potential off-target site 41088 for screening of mutants |
| 71_3`_41088_Off-Target | CAGGCTCCATGGTGATCACC | Amplification of potential off-target site 41088 for screening of mutants |
| 72_5`_41088_Seq | TCTCATTGCATGTATGCAAAGC | Sequencing primer for detection of mutations in potential off-target site 41088 |

**Suppl. Table 2:**

Oligos used to construct spacer sequences of gRNAs used in this study. The on- and off- target scores were calculated with the algorithms by (Doench et al. 2016) and (Hsu et al. 2013) respectively. A high on-target score implies a high probability for activity at the desired position, while a high off-target score suggests a low probability for off target effects. Lowercase letters represent overhangs of annealed adaptors with complementary overhangs for *Bsa*I digested ptCC9.

| construct | target score (on / off) | spacer sequence | forward adaptor | reverse adaptor |
| --- | --- | --- | --- | --- |
| VTC2-158 | 64,3 / 98,8 | GATCGCACGCGACATTCTGG | tcgaGATCGCACGCGACATTCTGG | aaacCCAGAATGTCGCGTGCGATC |
| VTC2-272 | 57,9 / 99,9 | GCCGCAAGCCGAGATTGTCC | tcgaGCCGCAAGCCGAGATTGTCC | aaacGGACAATCTCGGCTTGCGGC |
| PHO4-51 | 57,6 / 99,9 | GCCGTCCTCGGAGGTATCTG | tcgaGCCGTCCTCGGAGGTATCTG | aaacCAGATACCTCCGAGGACGGC |
| PHO4-980 | 62,4 / 99,0 | GCAGGGTGAAGTCTACGACC | tcgaGCAGGGTGAAGTCTACGACC | aaacGGTCGTAGACTTCACCCTGC |

**Suppl. Table 3:**

**Sequence of the predicted off-targets of VTC2-158 in Phatr2: ID42441 and Phatr2: ID41088.** Upper row shows the sequences used for *vtc2* editing. Deviations of the off-target sites to the on-target sites are highlighted in red.

| **Description** | **DNA sequence 5´->3´** |
| --- | --- |
| On-target site in *vtc2* (VTC2-158) | GATCGCACGCGACATTCTGG |
| Predicted off-target site in Phatr42441 | CATCGGACGCGAGATTCTGG |
| Predicted off-target site in Phatr41088 | GATCGCACGTTCCATTCGGG |

**Suppl. Figure 1: Scatterplot of the calculated CAFs for VTC2-272.**

The ordinate shows the position of the peaks relative to the position of the expected double strand breaks. -1 is defined as the position 4 bp upstream of the PAM and +1 is defined as the position 3 bp upstream of the PAM. The abscissae represents the calculated CAF for each position.


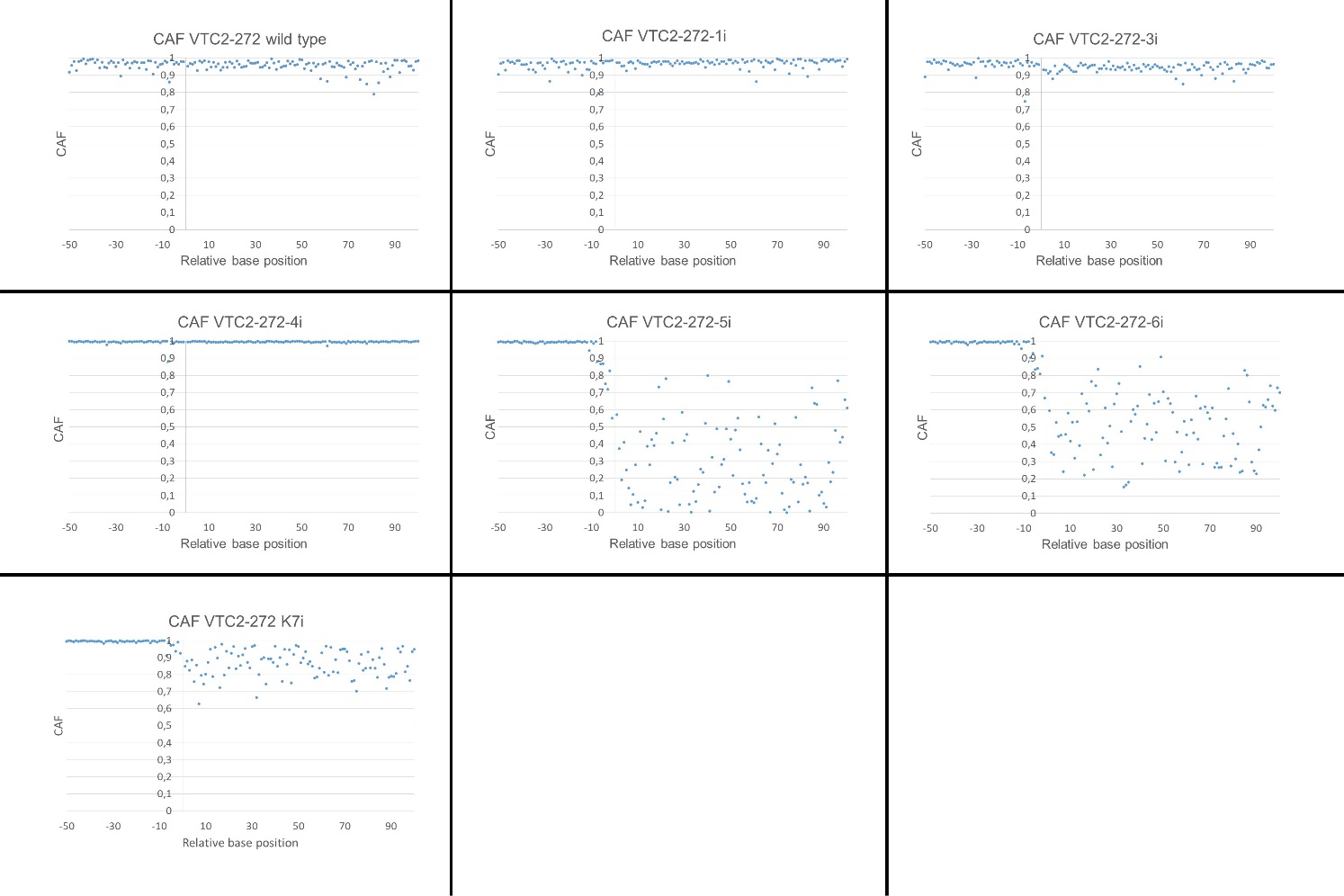


**Suppl. Figure 2: Chromatogram of the wild type *pho*4 gene and clones expressing ptCC9 with nucleotides 34-53 as the spacer of the *gRNA* gene (PHO4-51).** The arrow indicates the position of the expected DSB.

**Suppl. Figure 2 see next page**


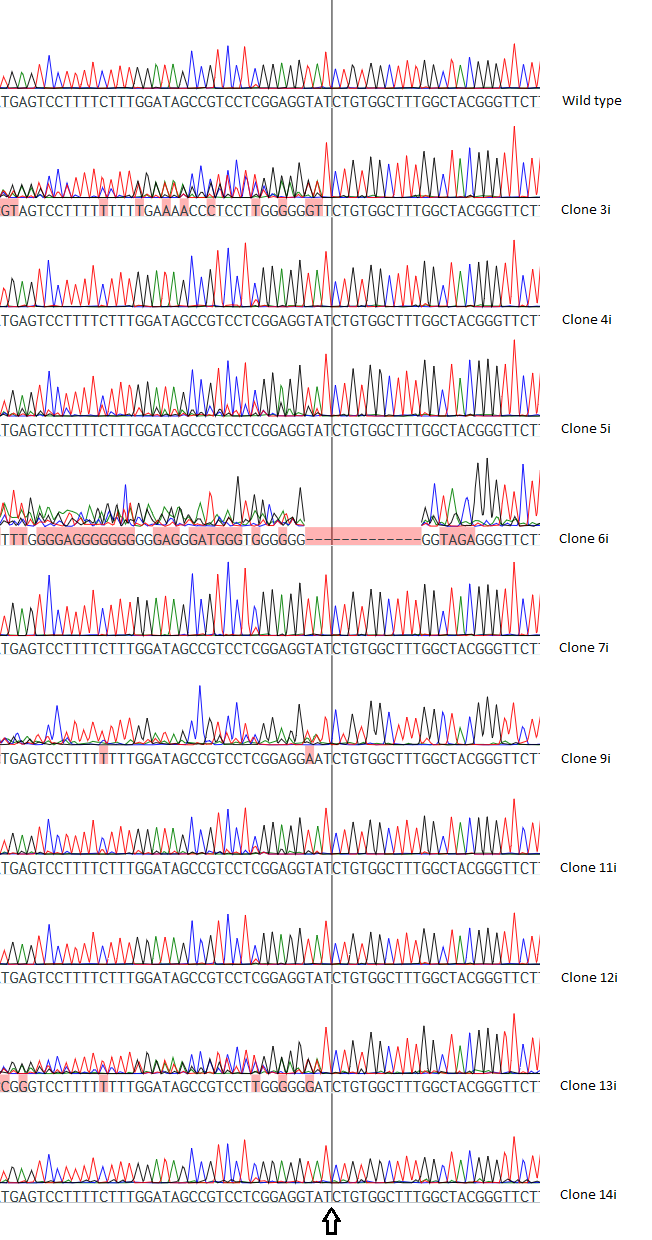


**Suppl. Figure 3: Chromatogram of the wild type *vtc*2 gene and *vtc*2 in VTC2-158 subclones.** The arrow indicates the position of the expected DSB.


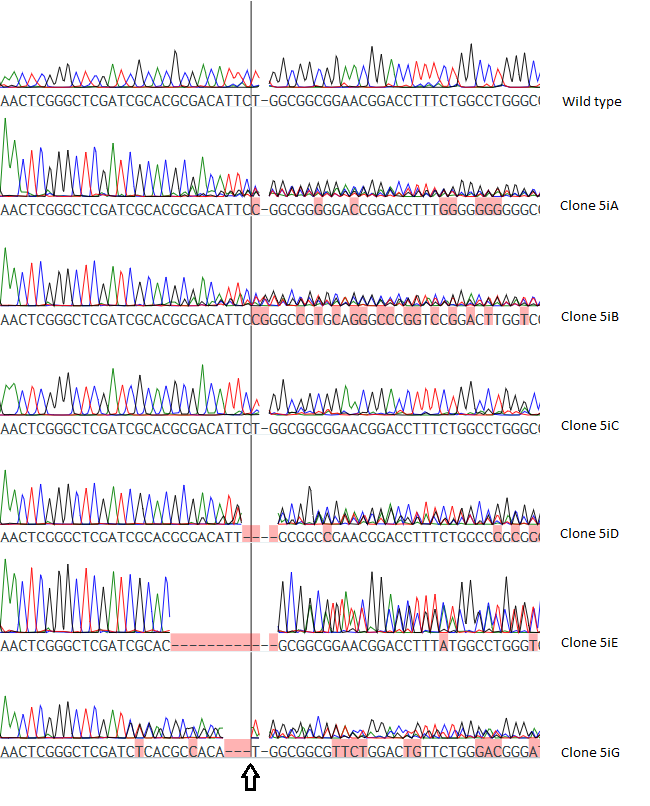


**Suppl. Figure 4: Chromatogram of the wild type *vtc*2 gene and *vtc*2 in VTC2-158 subclones.** The arrow indicates the position of the expected DSB.


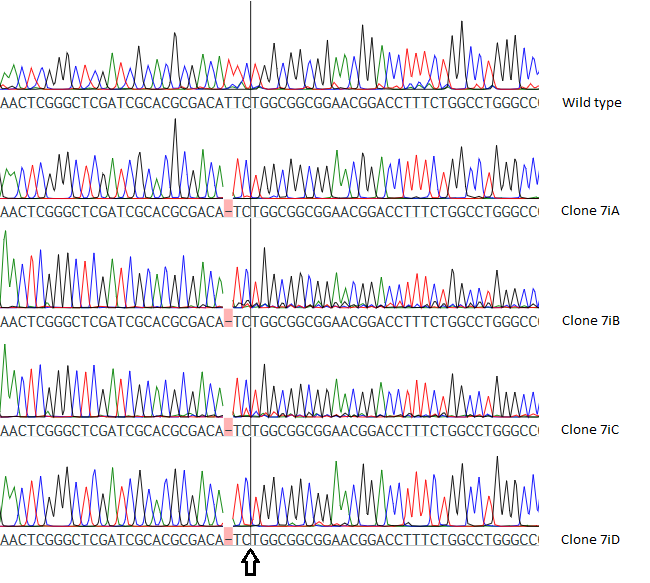


**Suppl. Figure 5: Chromatogram of the wild type *vtc*2 gene and *vtc*2 in VTC2-272 subclones.** The arrow indicates the position of the expected DSB.


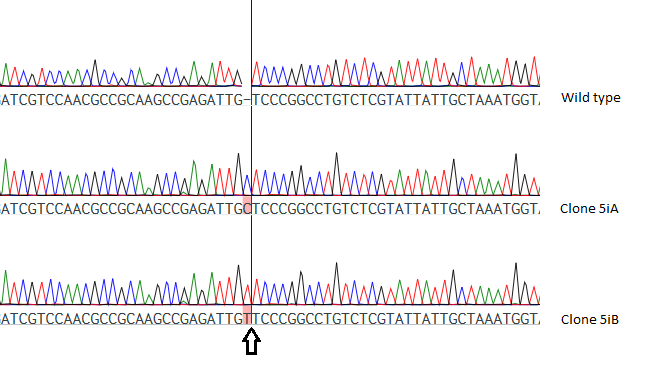


**Suppl. Figure 6: Chromatogram of the wild type *pho*4 gene and *pho*4 in PHO4-51 subclones.** The arrow indicates the position of the expected DSB.


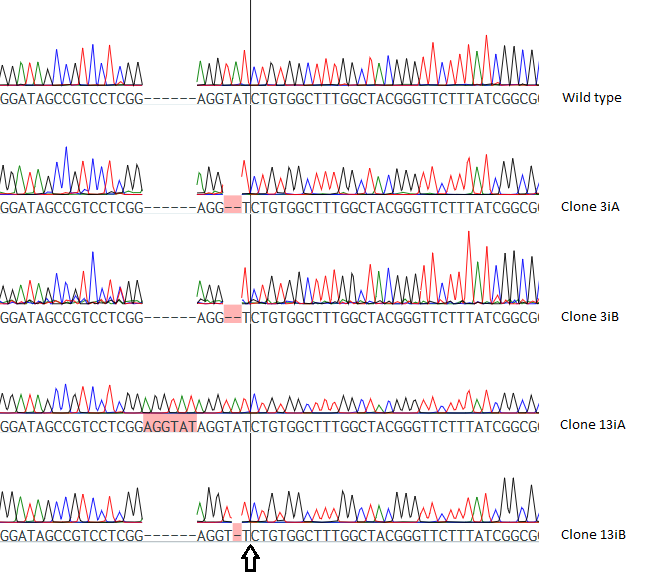
 **Suppl. Figure 7: Chromatogram of the wild type *vtc*2 gene and the PCR product amplified from VTC2-158-5E and cloned into pJET1.2/blunt Cloning Vector.** Single plasmids isolated from E. coli were used for sequencing. The arrow indicates the position of the expected DSB.


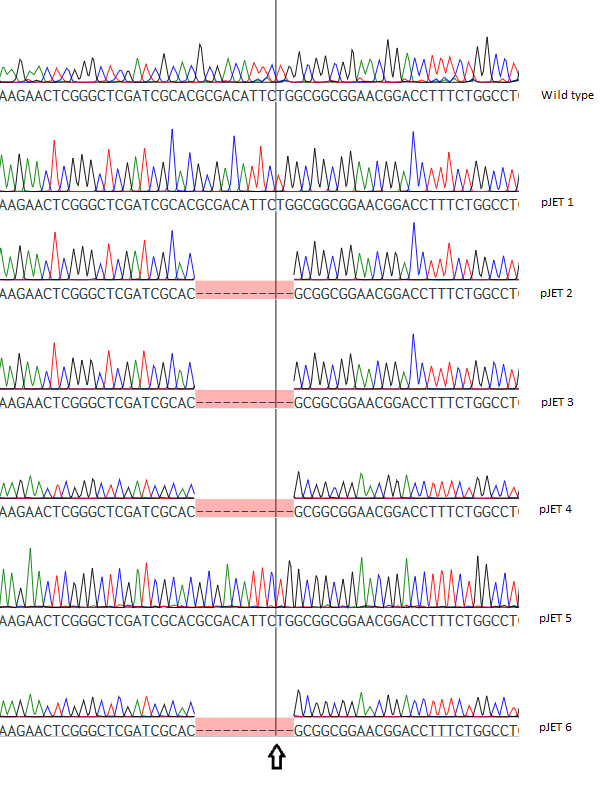


**Suppl. Figure 8: Chromatogram of the wild type *vtc*2 gene and the PCR product amplified from VTC2-158-5F and cloned into pJET1.2/blunt Cloning Vector.** Single plasmids isolated from E. coli were used for sequencing. The sequences pJET1, pJET5 and pJET6 showed the same 209 bp deletion. The arrow indicates the position of the expected DSB.


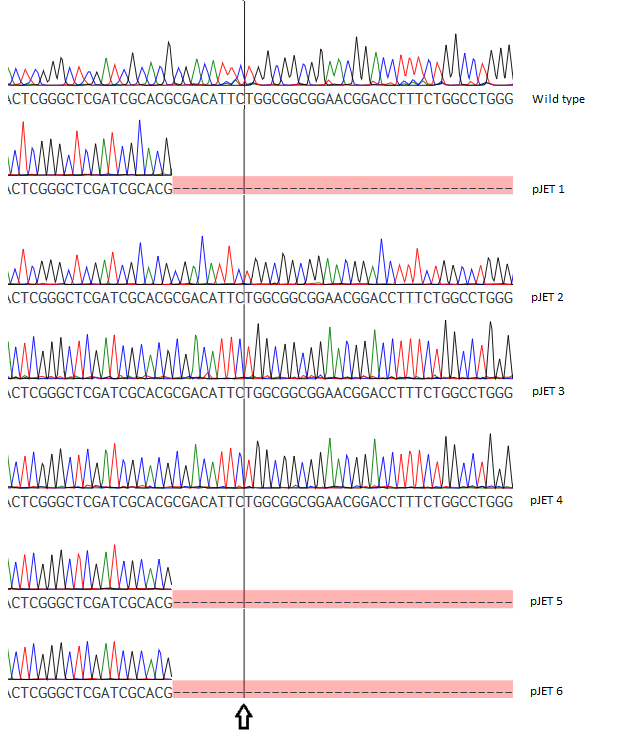


**Suppl. Figure 9: Chromatogram of the wild type *vtc*2 gene and the region of ID 41088, having a predicted off-target site.** The arrow indicates the position of the expected DSB.


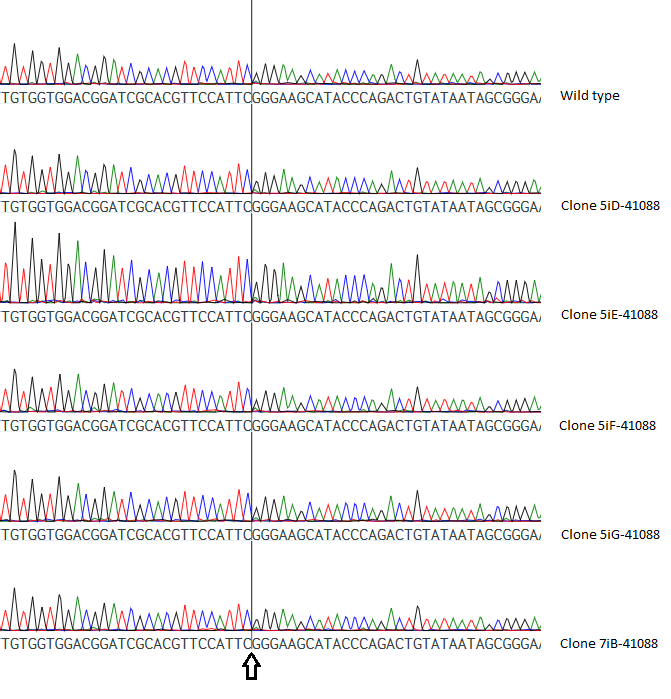


**Suppl. Figure 10: Chromatogram of the wild type *vtc*2 gene and the region of ID 42441 having a predicted off-target site.** The arrow indicates the position of the expected DSB.
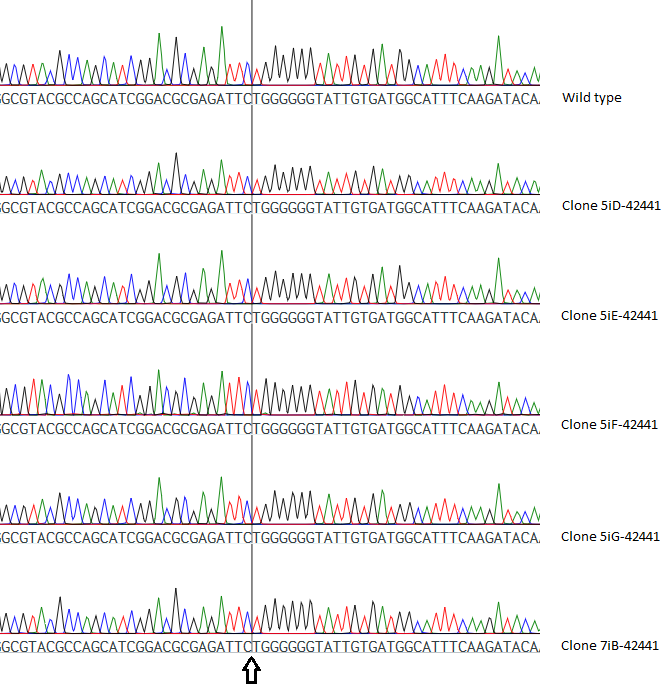

Supplement: Supplementary file 1 [file Data_Sheet_1.docx]
